# Complex general stress response regulation in *Sphingomonas melonis* Fr1 revealed by transcriptional analyses

**DOI:** 10.1038/s41598-019-45788-7

**Published:** 2019-06-28

**Authors:** Lisa Gottschlich, Petra Geiser, Miriam Bortfeld-Miller, Christopher M. Field, Julia A. Vorholt

**Affiliations:** Institute of Microbiology, Department of Biology, ETH Zurich, Vladimir-Prelog-Weg 1-5/10, 8093 Zurich, Switzerland

**Keywords:** Bacterial genetics, Bacterial transcription

## Abstract

The general stress response (GSR) represents an important trait to survive in the environment by leading to multiple stress resistance. In alphaproteobacteria, the GSR is under the transcriptional control of the alternative sigma factor EcfG. Here we performed transcriptome analyses to investigate the genes controlled by EcfG of *Sphingomonas melonis* Fr1 and the plasticity of this regulation under stress conditions. We found that EcfG regulates genes for proteins that are typically associated with stress responses. Moreover, EcfG controls regulatory proteins, which likely fine-tune the GSR. Among these, we identified a novel negative GSR feedback regulator, termed NepR2, on the basis of gene reporter assays, phenotypic analyses, and biochemical assays. Transcriptional profiling of signaling components upstream of EcfG under complex stress conditions showed an overall congruence with EcfG-regulated genes. Interestingly however, we found that the GSR is transcriptionally linked to the regulation of motility and biofilm formation via the single domain response regulator SdrG and GSR-activating histidine kinases. Altogether, our findings indicate that the GSR in *S. melonis* Fr1 underlies a complex regulation to optimize resource allocation and resilience in stressful and changing environments.

## Introduction

**Figure 1:**
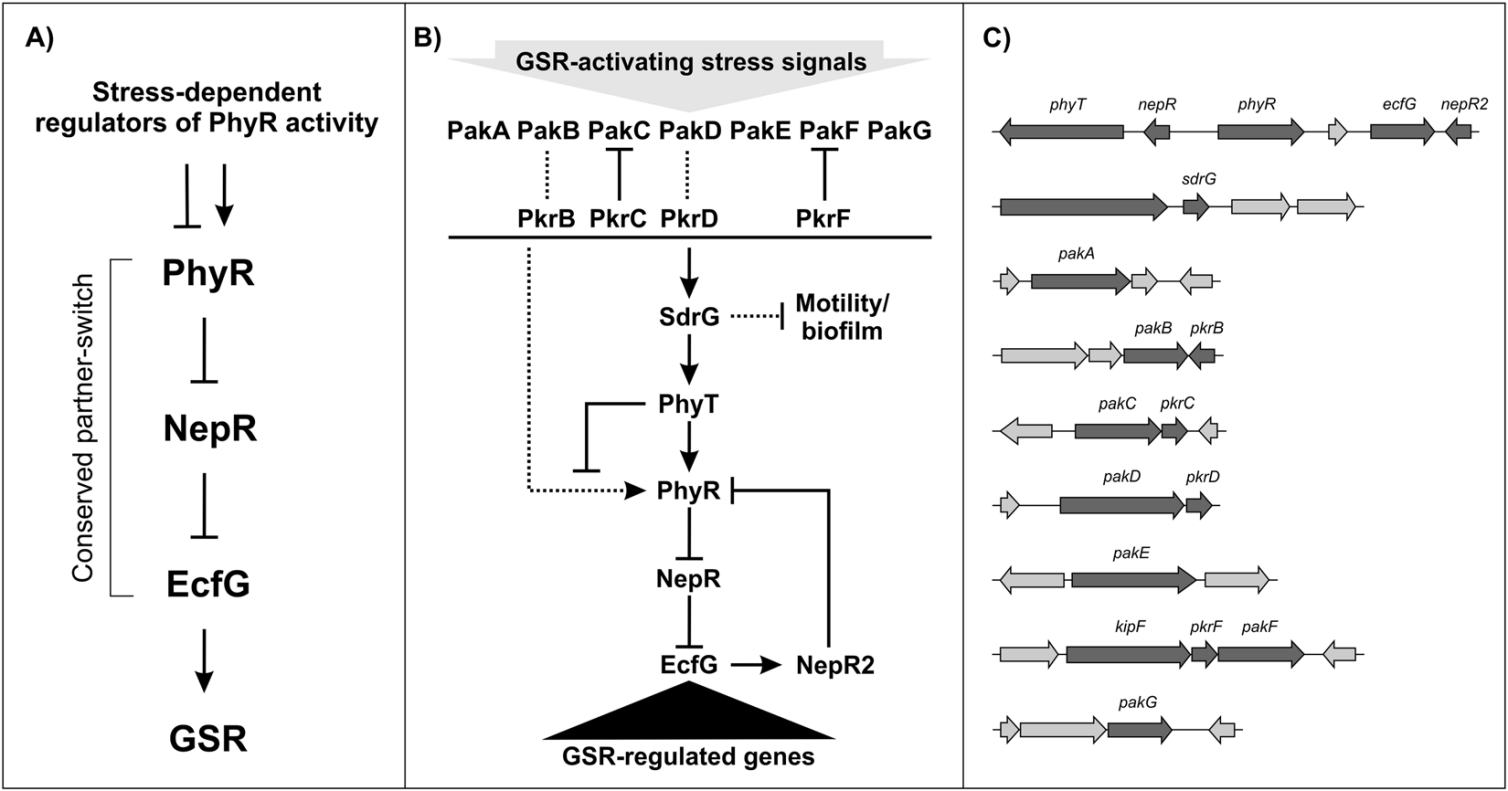
Regulation of the GSR. (**A**) Simplified version of the GSR regulation in alphaproteobacteria^1,2^. (**B**) GSR regulation in *S. melonis* Fr1. Activation of the GSR via the Pak-SdrG-PhyT-PhyR phosphorelay^42^ triggers the release of the alternative sigma factor EcfG via a partner-switching mechanism due to binding of the anti-sigma factor NepR to the phosphorylated anti-sigma factor antagonist PhyR^15^. EcfG binds to the RNA polymerase and activates transcription of the GSR-regulated genes. Direct PhyR phosphorylation by the Paks is represented by a dotted line, because it plays a minor role *in vivo*^42^. The role of NepR2 as a negative feedback regulator of the GSR and the connection of the response to motility and biofilm formation are also indicated. (**C**) Genes encoding GSR regulators in *S. melonis* Fr1.

Adaptability to fluctuating, stressful environmental conditions is a crucial trait for survival and fitness. In order to withstand a broad spectrum of stress stimuli, bacteria often utilize specific stress responses, as well as a general stress response (GSR) acting on a global level to provide cross-protection^1–4^. In general, the GSR integrates a variety of different environmental signals via sigma factor-mediated transcriptional control^5^. Stress-dependent alternative sigma factors compete with the housekeeping sigma factor for binding to the RNA polymerase to redirect transcription towards stress response genes. SigmaS is conserved in beta-, gamma- and deltaproteobacteria^6^ and has been studied in detail in *Escherichia coli*^3,7–9^, while SigmaB regulates the GSR in *Bacillus subtilis* and in other selected Gram-positive bacteria^10–12^. The alphaproteobacterial GSR is controlled by an ECF (extracytoplasmic function) sigma factor^1,2,13,14^ and has been analyzed for example in *Sphingomonas melonis* Fr1^15–18^, *Methylobacterium extorquens*^19–23^, *Bradyrhizobium diazoefficiens*^20,24,25^, *Sinorhizobium meliloti*^26–29^, *Brucella abortus*^30,31^, and *Caulobacter crescentus*^32–38^. These bacteria occupy different niches in nature and experience multiple stresses under environmental conditions but share core regulators of the GSR (Fig. 1A). This global stress response in alphaproteobacteria is triggered at the posttranslational level by protein-protein interaction after phosphorylation of the response regulator PhyR (phyllosphere-induced regulator) (Fig. 1A)^17,23,37^. The response starts with the direct or indirect activation of PhyR or its orthologues in other alphaproteobacteria by signal-integrating histidine kinases, which mainly belong to the HWE/HisKA2 family^4,16,18,28,31,35,39^. Upon phosphorylation at its receiver domain, the PhyR output domain becomes accessible. The latter resembles the ECF sigma factor and sequesters its anti-sigma factor, termed NepR (Fig. 1A). As a consequence of the partner switch, the alternative sigma factor EcfG is released and binds to the RNA polymerase to redirect transcription^17,23,37,40^. Despite the conserved GSR activation mechanism, different alphaproteobacteria may harbor paralogues of some of the core regulators, as is the case for *M. extorquens*^19^ and *S. meliloti*^27^.

As mentioned above, the activation of PhyR orthologues by kinases might be indirect. In fact, we recently showed that the phyllosphere commensal *S. melonis* Fr1^41^ harbors the bi-functional PhyR-phosphotransferase PhyT^42^, formerly PhyP^15^, which takes part in a GSR-activating phosphorelay together with the single domain response regulator SdrG^16,42,43^ (Fig. 1B,C); in addition, PhyT also prevents lethal overactivation of the GSR^15,42^. Notably, in a recent study, a GSR-activating phosphorelay has also been discovered in *C. crescentus*^35^, showing that such phosphorelays are more widespread than initially thought.

Several studies have provided knowledge about the transcriptional output of the GSR in different alphaproteobacteria^20,24,26,30–34,44,45^. Genes depending on the alternative sigma factor EcfG or its orthologues encode proteins that can, for example, be associated with envelope modulation, signal transduction, and stress protection^1,2^. Because the signaling cascade upstream of the GSR has been thoroughly investigated in *S. melonis* Fr1 and the alphaproteobacterium harbors only one EcfG paralogue, it provides an ideal model to analyze the GSR as a function of the upstream signaling proteins such as SdrG and the seven identified GSR-activating histidine kinases (Paks)^16^ after stress exposure. In the course of the study, we identified genes, which are regulated by EcfG and investigated stress-induced changes. The transcriptional analyses also indicated a counter regulation of the GSR with other cellular processes, i.e. motility and biofilm formation. In addition, we identified the negative GSR feedback regulator NepR2 (Fig. 1B,C).

## Results and Discussion

### Identification of EcfG-dependent genes under low stress conditions

Substantial knowledge has been acquired regarding the GSR-inducing signaling pathway in *S. melonis* Fr1, which involves the Pak-SdrG-PhyT-PhyR phosphorelay^15–18,42,43^ (Fig. 1B,C). However, little is known about the genes regulated by the GSR-controlling sigma factor EcfG. Here, we performed transcriptome analyses to identify the total number of genes whose expression is significantly influenced (directly or indirectly) by the alternative sigma factor (cutoff: log_2_ fold change ratio < [−1] and > 1, fdr < 0.05).

To characterize genes under EcfG control under low stress conditions, we first compared the transcriptomes of the Δ*ecfG* mutant and the wild-type strain grown in TYE medium, which has previously been shown to trigger only a low level of stress response using reporter gene assays^42^. Under this condition, we identified about 200 genes (Table S1), which represent about 5% of all *S. melonis* Fr1 genes.

As expected, most of the EcfG-regulated genes were downregulated in the Δ*ecfG* mutant compared to the wild type (Table S1). Several of these genes encode typical proteins associated with stress protection. In this respect, the differentially regulated genes include for example two catalases (#2489, #2832)^46^, a thioredoxin reductase (#2618)^47^, a peroxiredoxin (OsmC subfamily) (#2673)^48^, a NADH:flavin oxidoreductase (Old Yellow Enzyme family) (#2219)^49^, and two DNA-binding ferritin-like proteins (#1813, #2221)^50^, which are likely to play a role in oxidative stress protection. We also identified a ‘Ku’ protein-encoding gene (#1102), which is likely to be involved in DNA repair^51–53^. Our analyses also revealed EcfG-dependent genes, whose gene-products are known to be involved in the protection from salt and osmotic stress. These include amongst others small-conductance mechanosensitive channels (MscS) (#0242, #1832). In *B. subtilis* for example, the MscS-type YkuT is regulated by SigmaB and contributes to the protection to hypo-osmotic shock^54^. Transcriptional control of the osmoprotectant trehalose represents another strategy to cope with osmotic stress, as has for example been reported for the alphaproteobacteria *Rhodobacter sphaeroides* IL106^55^ or for *B. diazoefficiens*^24^, in which trehalose production is controlled by the GSR^24^. In *S. melonis* Fr1, the EcfG-dependent trehalose biosynthesis gene cluster (#1273-6) consists of a malto-oligosyltrehalose synthase-, a 4-alpha-glucanotransferase-, a malto-oligosyltrehalose trehalohydrolase-, and a glycogen debranching enzyme (GlgX)-encoding gene. Our analyses confirmed that the GSR core regulators PhyR, NepR and PhyT^15,42^, as well as a small number of additional regulators, including an orphan signal-sensing hybrid histidine kinase (#1746), depend on EcfG under low stress conditions.

We identified 33 genes that are upregulated in the Δ*ecfG* mutant compared to the wild type. These genes could be subject to indirect regulation by EcfG, for example via sigma factor competition^56,57^. One of the affected genes encodes a protein annotated as a membrane-bound Fe^2+^-dicitrate sensor (#1210), belonging to the ‘FecR superfamily’ (NCBI Conserved Domain Search). The genetic locus of this putative FecR-type regulator also includes a RNA polymerase sigma factor (sigma-70 family) (#1209) and a protein annotated as an outer membrane receptor for ferrienterochelin and colicins (#1208)^58,59^, suggesting that these proteins might participate in the regulation of iron homeostasis and therefore potentially counteract the production of reactive oxygen species^60^ during an activated GSR.

Classification of EcfG-regulated genes under low stress conditions with Clusters of Orthologous Groups (COG) categories^61^ retrieved from the Integrated Microbial Genomes with Microbiome Samples system (IMG/M: https://img.jgi.doe.gov/m/)^62^ (Fig. S1, Table S2), revealed that the GSR also causes an adaptation of more general processes like metabolism, transport, and envelope modulation. In addition, we found that about 40% of the EcfG-dependent genes cannot be associated with a specific function, revealing the potential to discover novel and possibly critical functions of the GSR in *S. melonis* Fr1.

Altogether, our data suggest that the GSR in *S. melonis* Fr1 adjusts multiple cellular pathways by controlling a core set of genes under low stress conditions, which enable a rapid counteraction as a first line of defense until the enhanced and full response is mounted.

### Identification of putative EcfG-binding sites

**Figure 2:**
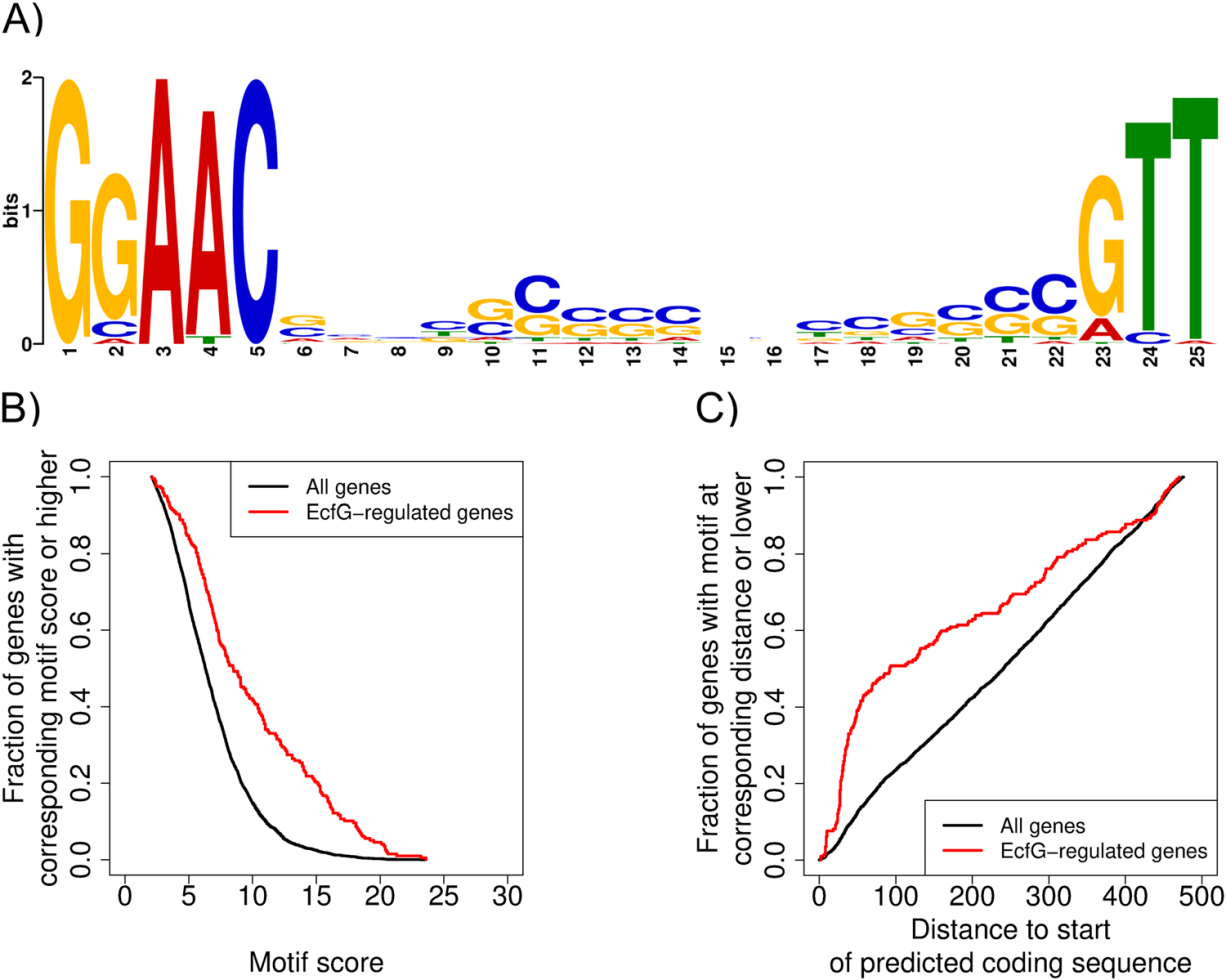
Search for direct target genes of EcfG. (**A**) A novel EcfG-binding motif more specific for *S. melonis* Fr1 was created to identify direct targets of the alternative sigma factor. For details see Experimental Procedures. (**B**) Putative EcfG-binding motifs can be identified throughout the genome, but high scoring motifs accumulate upstream of genes regulated by EcfG. A higher scoring motif is more likely to represent a direct target for the alternative sigma factor. (**C**) The best-scoring motifs for the EcfG-regulated genes accumulate within a distance of 100 bp to the start of the predicted coding sequence.

Because the regulation by the alternative sigma factor EcfG can be direct or indirect, we conducted an EcfG-motif search using the GLAM2Scan software^63^, which is part of MEME Suite^64^, as described in Experimental Procedures. Based on the 50 genes with the best scoring motifs within the genes differentially regulated in an EcfG-dependent manner, we constructed the gapped EcfG-binding motif GGAAC-N_17–20_-GTT (Fig. 2A). As expected, the motif matches the consensus proposed to be recognized by EcfG alternative sigma factors^13,15,19,20,24,26,32,65^, but is more specific for *S. melonis* Fr1.

We used this motif to identify putative EcfG-binding sites throughout the *S. melonis* Fr1 genome. This analysis revealed that potential EcfG-binding sites, corresponding to the best-scoring identified motifs (Table S3), occur in the upstream region of the start of the predicted coding sequences (CDS) throughout the whole genome, but accumulate upstream of genes that were regulated in an EcfG-dependent manner (Fig. 2B). Based on a direct comparison of the upstream regions of the positively EcfG-controlled genes with the motif GGAAC-N_17–20_-GTT, we decided to use a motif score cutoff value of 10 to differentiate between potential direct and indirect targets of the alternative sigma factor (Table S3). It also became obvious that the motifs of the EcfG-controlled genes seem to be enriched within the first 100 bp upstream of the start of the predicted CDS (Fig. 2C).

From this we conclude that all of the 166 positively EcfG-regulated genes with a matching motif located at a distance of up to 100 bp upstream of the start of the predicted CDS are likely to be direct targets of the alternative sigma factor, which includes about 35% of the respective genes under low stress conditions (Table S3). If we include genes, which are potentially encoded in operons with putative direct EcfG target genes, this percentage even increases. However, the number of EcfG targets might still be underestimated, since genes with a matching motif, which is located further upstream than 100 bp from the start of the predicted CDS could also represent potential direct targets of the alternative sigma factor. The motif scores and the distances to the start of the predicted CDS of the 50 genes with the most severe transcriptional differences between the Δ*ecfG* mutant and the wild-type strain, only focusing on genes with an apparent positive regulation by EcfG, are visualized in Table 1.

**Table 1:** The top positively EcfG-regulated genes under low stress conditions.

Transcript ID	Predicted function	Sequence of putative EcfG-binding motif	Distance to start of the predicted CDS	Motif score	log_2_ ratio
#0072	‘cAMP-binding proteins - catabolite gene activator and regulatory subunit of cAMP-dependent protein kinases’	**GGAAC**GTTACGCCCCCGATCCGT**GTT**	10	13.9	−3.5
#0098	‘hypothetical protein’	**GGAAC**AATGCGGGGCCCGTGCGC**TTT**	29	14.1	−3.2
#0597*	‘probable methyltransferase’	GGAACGGCAGGACGGCGTGTGGACGCGCT	448	6.03	−3.9
#0598	‘TIGR03440 family protein’	GCAACTCTTTGAGCTGTCATGCGCT	52	6.61	−4.0
#0628	‘Outer membrane protein V'	**GGAAA**GATTCCGCAACCGCTGC**GTT**	76	11.9	−3.3
#0787	‘hypothetical protein’	**GGAAC**GATAGCGGAAACAGGCG**GTT**	54	18.1	−3.2
#1028	‘Uncharacterized protein conserved in bacteria’	GCACCTCAGGCGGCGCCTCCGCCCGTT	448	8.49	−3.3
#1060	‘hypothetical protein‘	**GGAAC**AAGCGGTCGCAGCCTGTG**GTT**	41	15.4	−3.9
#1102	'Ku protein, prokaryotic'	**GGAAC**GACTCTTTCTCCCATGA**ATT**	30	10.2	−3.2
#1274*	‘4-alpha-glucanotransferase’	CGATCCGCAGGCGCGCGCACGCATT	311	6.55	−3.5
#1275*	malto-oligosyltrehalose trehalohydrolase'	GGCAGAACCCCGAAGGCCGCGCGCT	234	2.97	−3.3
#1276	‘glycogen debranching enzyme GlgX’	GGGACCTTCCATGCTGCGCCTGCGTT	167	5.11	−3.1
#1281	‘Predicted outer membrane protein’	**GGAAC**GCAGCGCCGCGTCCGAAC**GTT**	27	16.1	−7.3
#1282	‘Domain of unknown function (DUF3597).’	GGAGCATTCGGCGCGCATCGACGTT	47	7.8	−3.9
#1297	‘Aerobic-type carbon monoxide dehydrogenase, large subunit CoxL/CutL homologs’	GCAACCCGGCGGTGGAGGCCGCTTT	151	9.05	−3.3
#1298*	'Aerobic-type carbon monoxide dehydrogenase, middle subunit CoxM/CutM homologs'	GGCACGCCCGACGACCTCCACCCGCT	294	8.05	−4.5
#1299	‘Aerobic-type carbon monoxide dehydrogenase, small subunit CoxS/CutS homologs’	GGAACGGATGCCAGCAGCATCCCGGT	93	8.84	−4.8
#1442	‘Predicted small secreted protein’	**GGAAC**GAATGGCCCGACCGATC**ATT**	30	16.9	−4.7
#1444	‘hypothetical protein’	**GGAAC**CAACGAACGCGACTTTG**GTT**	92	10.9	−3.4
#1446	‘hypothetical protein’	GGACCCAAAACGGGTTTCGGGGGTT	2	9.76	−6.2
#1462	‘Uncharacterized protein conserved in bacteria’	GCATCCATTTGGAACACGGGTCGTT	31	7.33	−6.1
#1478	‘PRC-barrel domain.’	**GGAAC**CGGCGCGGCCACAAGGCG**GTT**	27	18.1	−4.5
#1484	‘hypothetical protein’	CGAACTCGTGGCCCGCGTCAGCGCGGT	444	3.54	−5.1
#1611*	‘Raf kinase inhibitor-like protein, YbhB/YbcL family’	GCGACGAACGCATCCGCCGCCGATT	240	8.51	−3.2
#1612	‘hypothetical protein’	**GCAAC**CGCCGGGGCCTGTCTTGC**GTT**	2	15.9	−3.5
#1750	‘Glycosyltransferase’	**GGAAC**AACTCCGCCACGCCGGA**GTA**	69	12.7	−3.8
#1751	‘Glycosidases’	**GGAAC**CCCCTCGCCCCTCCTCC**GTT**	26	21.3	−3.4
#1765	‘NAD-dependent aldehyde dehydrogenases’	**GGATC**GCGTGCCGGGCCTGCGC**GTT**	8	19.2	−4.4
#1831	‘Topoisomerase IB’	**GGAAC**CGTGCGCGTCGCCTGGGC**ATT**	24	17.9	−3.5
#1836	‘Uncharacterized stress protein (general stress protein 26)’	**GCAAC**CAACCCCCGATGCGCTC**GTT**	34	16.7	−4.2
#1930	‘hypothetical protein’	**GGAAC**CCTTACCGATGCACGCC**GTT**	154	18.3	−4.8
#2026	‘hypothetical protein’	**GGAAC**TTCGGTCCAAATGGTGC**GTT**	35	13.1	−6.1
#2180	‘hypothetical protein’	**GGAAC**CGCCGAGCGGATCGCGC**GTT**	270	20.4	−6.3
#2452	‘hypothetical protein’	GATACGCCCGCGATCCCAACGCGTT	28	7.71	−3.6
#2489	‘Catalase.’	CGATCGGCTGCGGCATCTGCGCATT	296	8.83	−3.7
#2499	‘Zn-dependent alcohol dehydrogenases’	GGCACGGCGCTGTGCGACGCCGGCT	470	6.61	−3.2
#2509	‘hypothetical protein’	GAAACGCCCGCCCAGCCCAGCCAGCCGGT	450	4.68	−4.3
#2510	‘hypothetical protein’	**GCAAC**CCGCTGGGCTGCGGGAC**GTT**	25	14	−4.1
#2527	‘Outer membrane protein’	**GGATC**AACCGCGGGGGACGGCC**GTT**	440	18.4	−4.7
#2528*	‘Uncharacterized protein conserved in bacteria’	GCGACGCGCTGGCGCCATCGCGCCGCT	337	6.02	−4.3
#2534	‘hypothetical protein’	**GGAAC**GGCTCCATTCCTGACCA**GTT**	8	12.5	−4.2
#3063	‘Protein required for attachment to host cells’	**GCAAC**CCGCCCGCCTCGGCTGC**GTT**	34	16.9	−3.9
#3142	‘hypothetical protein’	**GGAAC**CGGTATCGCCTGCGTAAC**GTT**	49	10.7	−3.3
#3266	‘Uncharacterized conserved protein’	GGTGCGCCGGCGTGCGCTCGCCGCT	157	3.33	−3.2
#3278	‘hypothetical protein’	**CGAAC**CGTTTCCCCCGTCCGCTC**GTT**	28	14.2	−3.2
#3404	‘Predicted integral membrane protein’	GGAACAGATGCTGGCCCAGCGCCTCGTC	308	6.8	−3.3
#3506*	‘Predicted glycosyl transferase’	GCCACGCCCCGGCCTGCTGCGCGTG	92	6.27	−3.3
#3507*	‘Predicted glycosyltransferases’	GGCTCGAAGCGCTCGCCGACCGGTT	51	6.97	−3.4
#3508*	'Glycosyltransferase'	**GAAAC**CTTCCTGCGCGTCGGCG**GCT**	400	11.3	−3.7
#3509*	‘Exopolysaccharide biosynthesis protein’	GGTCGTCGGCGACGCCGGCCGGTT	171	5.26	−4.3

The table indicates transcript IDs (refering to Sphme2DRAFT numbers), predicted function, sequence of the putative EcfG-binding motif, distance (number of nucleotides) of the putative motif to the start of the predicted coding sequence (CDS), score of the identified putative EcfG-binding motif according to GLAM2^63^ (see also Experimental Procedures) and log_2_ fold change ratios of the top 50 genes regulated in an EcfG-dependent manner under low stress conditions, i.e. the bacteria were grown in TYE medium without exposure to other stress stimuli (cutoff: log_2_ fold change ratio < [−1] and > 1, fdr < 0.05). Putative -35 and -10 promoter elements are highlighted within motifs with a score above the cutoff ≥ 10. Note, EcfG is not included in the list because its log_2_ fold change ratio is due to its knockout. *Genes overlap with the previous gene/s and are therefore likely encoded in an operon; these genes are unlikely to harbor an active EcfG-binding motif.

**Figure 3:**
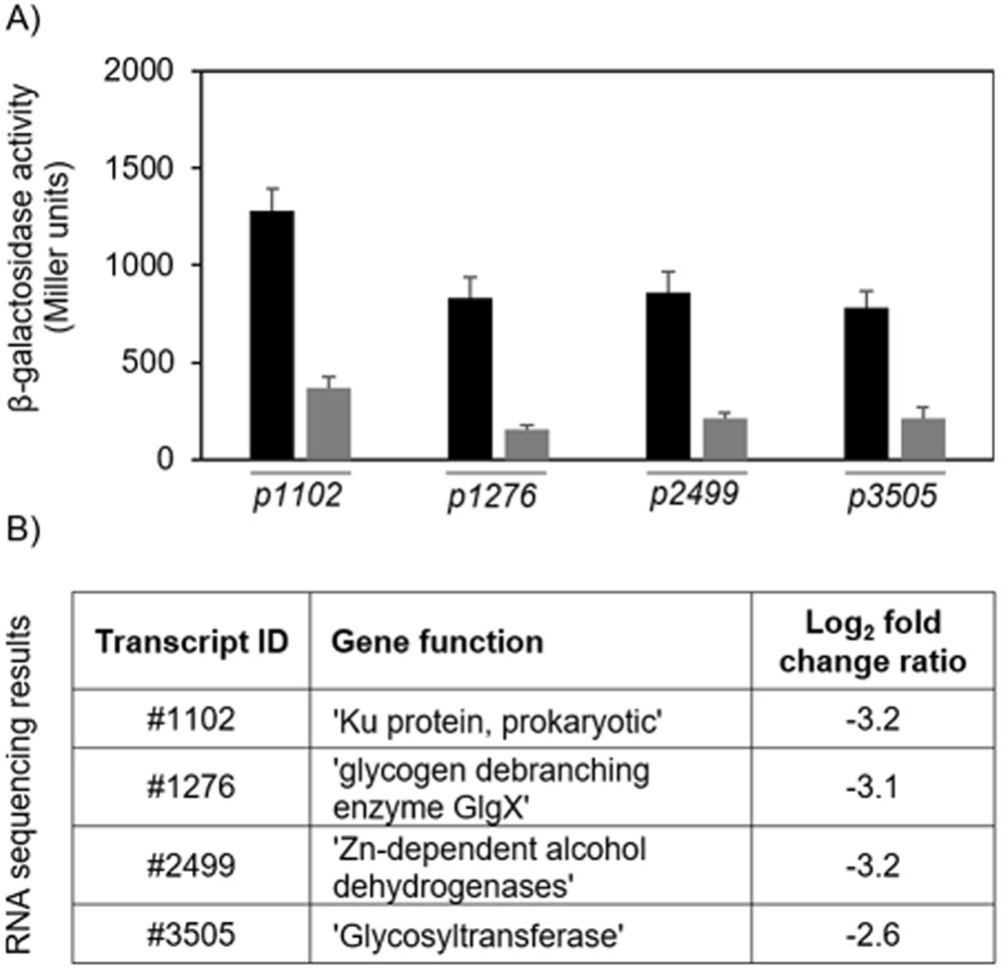
*In vivo* confirmation of the RNA sequencing results. (**A**) β-Galactosidase activity of different *promoter*-*lacZ* fusions in *S. melonis* Fr1 wild type (black bars) and the Δ*ecfG* mutant (grey bars) under low stress conditions (TYE medium) to confirm dependency of the corresponding genes on EcfG. Values are given as mean ± SD of three independent experiments. (**B**) Transcript IDs, gene functions, and log_2_ fold change ratios of the corresponding genes resulting from the RNA sequencing data relating to the Δ*ecfG* vs wild type comparison under low stress conditions are indicated; the false discovery rate (fdr) is below 0.05 for all listed genes. Log_2_ fold change ratios were rounded to one decimal place.

We successfully validated the EcfG-dependency of genes identified in the course of the transcriptome analyses with β-galactosidase assays (Fig. 3), testing the upstream region of a ‘Ku’ protein-encoding gene (#1102) as a potential direct target and the upstream regions of a glycogen debranching enzyme (GlgX)- (#1276), a Zn-dependent alcohol dehydrogenase- (#2499) and a glycosyltransferase-encoding gene (#3505) as putative indirect targets of the alternative sigma factor EcfG.

### Adaptation of the GSR after exposure to a stress mix

Next, we investigated the GSR of *S. melonis* Fr1 in response to a complex stress stimulus, ensuring activation of multiple stress-integrating Paks^16^. Therefore, we applied a stress mix inducing salt (80 mM sodium chloride), ethanol (1% ethanol), and oxidative stress (50 µM tert-butyl hydroperoxide), which was already used in a previous study to activate the GSR in *S. melonis* Fr1^42^. First, we performed β-galactosidase assays (Fig. S2) with the EcfG-dependent *nhaA2p*-*lacZ* reporter^16^ after encountering this stress mix or the corresponding individual stress stimuli. Indeed, we could validate GSR activation under the tested conditions.

**Figure 4:**
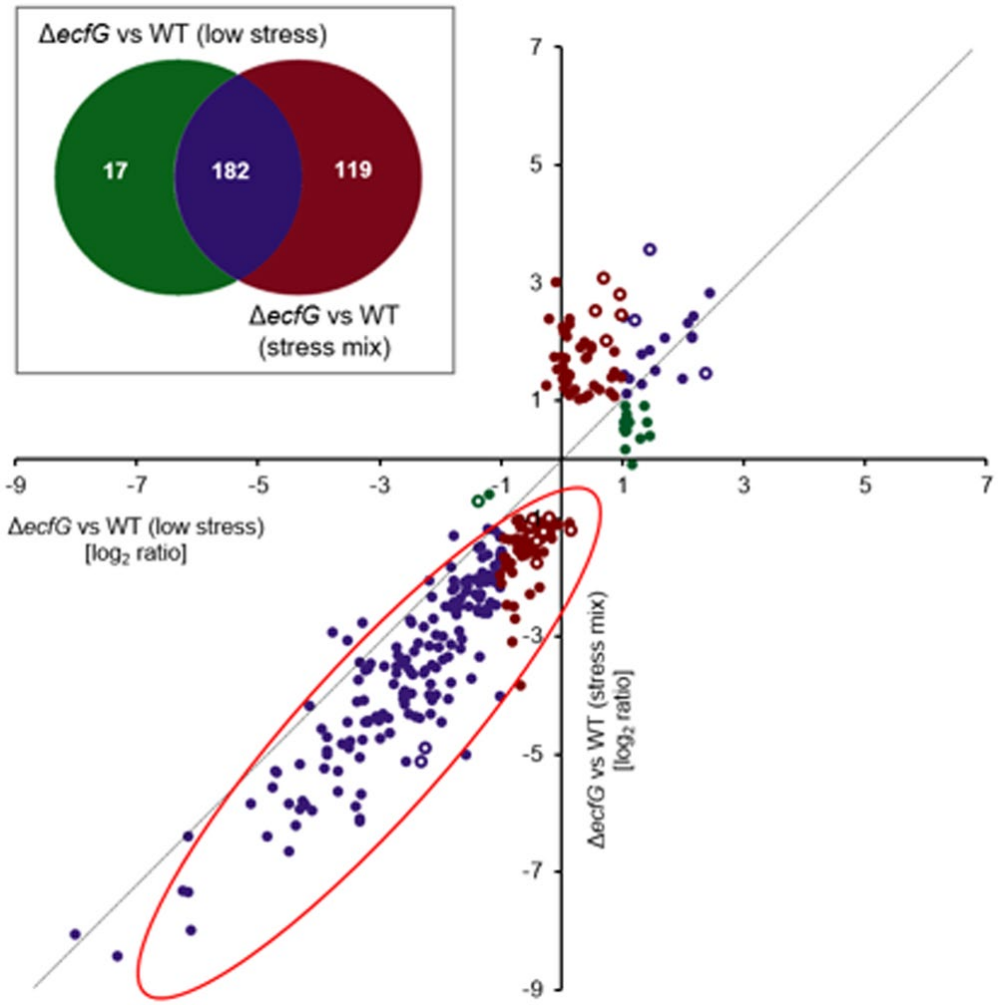
Impact of stress mix exposure on the expression of EcfG-regulated genes. The Venn diagram and the scatter plot were generated based on the UGent Venn diagram web tool (http://bioinformatics.psb.ugent.be/webtools/Venn/). The genes regulated in an EcfG-dependent manner under low stress conditions in *S. melonis* Fr1 (Δ*ecfG* vs WT) and 1 h after exposure to a stress mix (80 mM sodium chloride, 1% ethanol, 50 µM tert-butyl hydroperoxide) (cutoff: log_2_ fold change ratio < [−1] and >1, fdr < 0.05) were used for this analysis (Table S4). The white numbers describe the significantly regulated genes. The scatter plot displays the log_2_ fold change ratios of the genes represented in the corresponding Venn diagram. Green means “exclusively regulated by EcfG under low stress conditions”, purple means “regulated by EcfG under both conditions” and dark red means “exclusively regulated by EcfG 1 h after exposure to the stress mix”. The red circle indicates that the genes regulated by EcfG under low stress conditions are more strongly induced in the Δ*ecfG* mutant vs wild type comparison after stress mix exposure. The open circles in the scatter plot indicate genes that are transcriptionally regulated in the Δ*ecfG* mutant. The transcript IDs of the corresponding genes are highlighted in yellow in Table S4.

The results of the transcriptome analyses of the wild-type strain and the Δ*ecfG* mutant revealed an adaptation to the encountered stress mix (Fig. 4, Table S4). The majority of genes that were significantly downregulated in the Δ*ecfG* mutant compared to the wild type under the low stress conditions showed an increased differential transcription (expressed as log_2_ fold change ratios) after exposure to the stress mix (Fig. 4, scatter plot, red circle). As expected, this shift in log_2_ fold change ratios was mainly based on an upregulation of the respective genes in the wild type (Table S4), which is likely to underlie an increased level of free EcfG after the stress exposure, but which could also be influenced by stress-dependent additional regulators in the wild type. From these results we conclude that EcfG contributes to a pre-adjustment of the bacteria to potential future stress encounters under low stress conditions.

In contrast to 17 genes that were significantly regulated only under low stress conditions, the number of genes under EcfG control extended to an additional set of 119 genes that were significantly regulated after exposure to the stress mix (Fig. 4, Venn diagram), of which about 40% could not be associated with a specific function. In total, 73 out of these 119 genes were significantly downregulated in the Δ*ecfG* mutant compared to the wild type. In addition to several genes associated with general processes such as metabolism, transport, and envelope modulation, we also found stress protection associated protein-encoding genes. These candidates were mainly related to oxidative stress protection, including a glutathione S-transferase (#2403)^66^, a NADH:flavin oxidoreductase (Old Yellow Enzyme family) (#0186)^49^ and a catalase/peroxidase (HPI) (#2722)^67^. The genes significantly regulated in response to the stress mix also included additional signal transduction protein-encoding genes, including two single domain response regulators^68^ (#2801, #0512), of which the latter has been identified as PkrB and is associated with the GSR-activating histidine kinase PakB (#0513)^16^. In contrast to EcfG-dependent gene regulation in the wild type after exposure to the stress mix, we also identified a small number of genes (18 in total) which are transcriptionally regulated in the Δ*ecfG* mutant. These genes are indicated by open circles in the scatter plot in Fig. 4.

Exposure to the stress mix resulted in 46 additional genes significantly upregulated in the Δ*ecfG* mutant compared to the wild type. In addition to many genes encoding proteins associated with metal transport, we identified another putative FecR-type regulator encoded next to a RNA polymerase sigma factor (sigma-70 family) (#2100-1). As mentioned above, this apparent negative regulation by EcfG after stress mix exposure might be caused by sigma factor competition^56,57^ and might reflect a further adaptation to lower the risk of production of reactive oxygen species under stress conditions^60^.

In addition to the genes regulated in an EcfG-dependent manner, we observed downregulation of multiple genes encoding proteins associated with motility and biofilm formation 1 h after exposure to the stress mix in the Δ*ecfG* mutant, but also in the wild type (visualized in cluster III of the heat map in Fig. S3, Table S5). These genes include Flp pilus- and Flagellin-encoding genes, as well as genes encoding c-di-GMP and chemotaxis regulation-associated proteins. Interestingly, exposure to the individual components of the stress mix does not induce the observed downregulation (cluster III in Table S5). Further analysis revealed that this EcfG-independent regulation is dependent on the GSR key regulators SdrG and the Paks (see below).

### Congruency and divergence of gene regulation by the GSR key regulators

**Figure 5:**
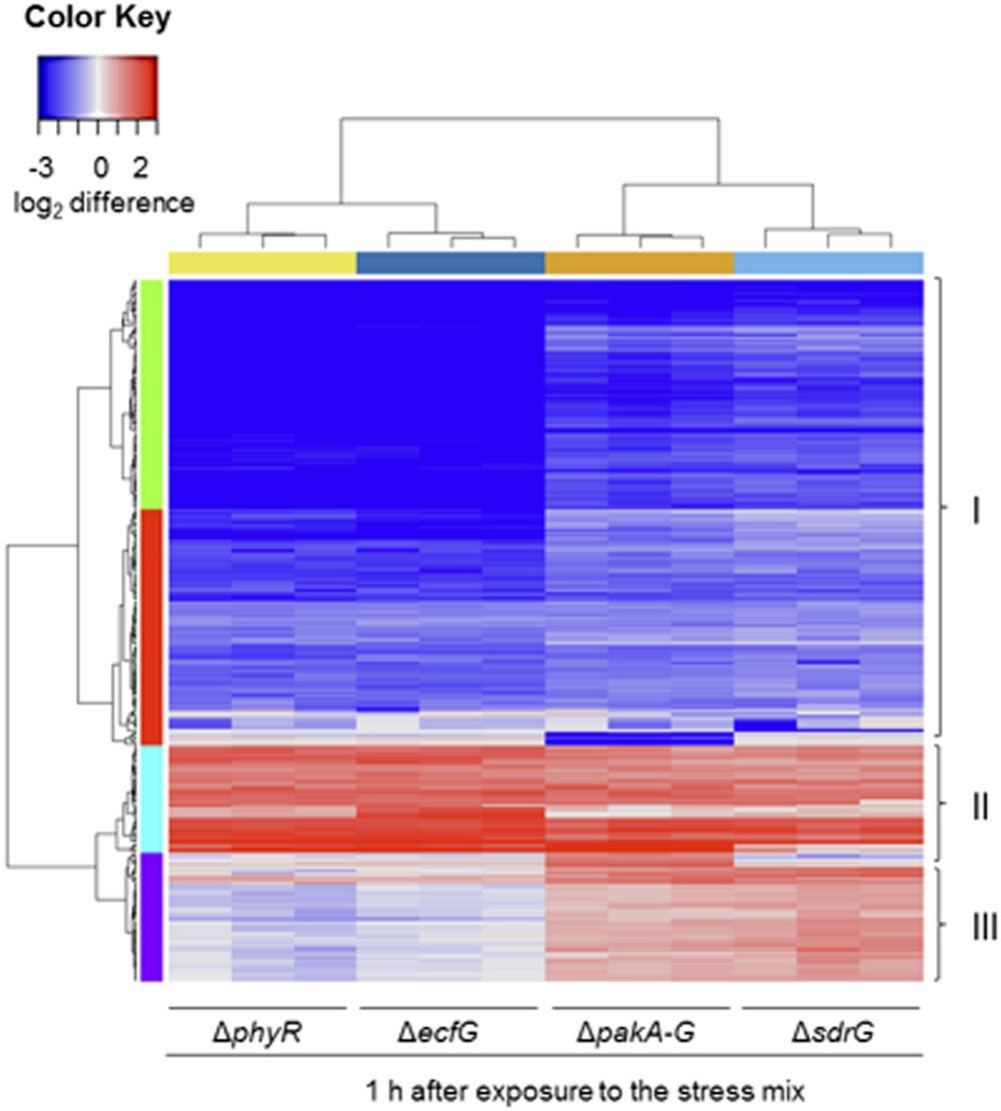
Comparison of the transcriptomes of PhyR, SdrG, and the Paks with that of EcfG. Heatmap including the 300 most variable genes in the GSR key regulator knockout mutants (Table S6) normalized to wild type 1 h after exposure to the stress mix (80 mM sodium chloride, 1% ethanol, 50 µM tert-butyl hydroperoxide), which is not shown. Clusters I and II represent genes which are equally regulated in all GSR key regulator knockout mutants. Cluster III contains mainly motility- and biofilm formation-associated genes. The majority of these genes are upregulated in the Δ*sdrG* and the Δ*pakA-G* mutants compared to the wild type, as well as to the Δ*phyR*, and the Δ*ecfG* mutants after exposure to the stress mix. The heatmap was generated with the function heatmap.2 from the R-package “gplots” (version 3.0.1). The image represents three independent biological replicates.

In order to obtain additional information on the regulatory impact of PhyR, SdrG, and the Paks (Fig. 1B,C) on the GSR, we analyzed the transcriptomes of a Δ*phyR*, a Δ*sdrG*, and a complete Δ*pak* mutant after exposure to the stress mix and compared them to that of the Δ*ecfG* mutant. This comparison is visualized in the heat map in Fig. 5 (Table S6), which is normalized to the wild type. We found 304 genes that were differentially regulated by PhyR compared to 301 genes that are under EcfG control under the tested condition (Fig. S4, Table S7). The number of overlapping genes was 282. Additionally, a small group of 22 genes was only significantly regulated in the Δ*phyR* mutant, while 19 genes were only identified to be significantly regulated in the Δ*ecfG* mutant. The substantial overlap of the genes regulated by PhyR and EcfG, respectively, underlines the tight partner-switching mechanism triggered by PhyR^23^. We found that *ecfG* is downregulated in the Δ*phyR* mutant. This suggests that the alternative sigma factor is autoregulated similarly to EcfG orthologues in other alphaproteobacteria^24,26,32,44,45^ and many ECF-type sigma factors in general^13,14,65,69–72^.

The tested GSR key regulators, EcfG, PhyR, SdrG, and the Paks, have 219 significantly regulated genes in common (Fig. S4, Table S7). This congruence is also evident in the heat map of regulated genes shown in Fig. 5 (clusters I and II). However, we observed that a distinct group of genes, of which many are linked to motility and biofilm formation regulation, is upregulated in the Δ*sdrG* and the Δ*pak* mutants compared to the wild type, the Δ*ecfG*, and the Δ*phyR* mutants (cluster III). Interestingly, numerous of these genes had indeed been identified as downregulated in the wild type after exposure to the stress mix (cluster III in Fig. S3, Table S5); see also Table S8. These transcriptome data imply that the level of phosphorylated SdrG, as a target of the Paks^16,42^, might play a key role in counter regulating the GSR with motility and biofilm formation under severe stress conditions. Interestingly, it was also shown that the SdrG orthologue MrrA from *C. crescentus* has an inhibitory function on motility and chemotaxis^35^. This finding further corroborates our results regarding the role of SdrG in the regulation of motility and biofilm formation. Overall, this uncovered regulatory link may provide an important measure to prioritize investment in protein synthesis under strong GSR-inducing environmental conditions^73–75^.

### Identification of the novel negative feedback regulator NepR2

The above results highlight the link of EcfG regulation and additional regulatory circuits. Controlling the level of EcfG available for binding to the RNA polymerase helps to keep the balance between beneficial stress response and lethal overactivation of the GSR^15,26^. To ensure such a precise adjustment, the GSR underlies transcriptional feedback control, for example mediated by the anti-sigma factor NepR (#1444)^15^ or the PhyR phosphotransferase PhyT (#1443) in *S. melonis* Fr1^15,42^. In the course of this study, we identified #1448 as part of the EcfG regulon, which is located next to *ecfG* (#1447) in the genome of *S. melonis* Fr1 (Fig. 1C). Transcription of the gene is increased after stress mix exposure in an EcfG-dependent manner in the wild type, although this upregulation is slightly below our significance cutoff (log_2_ fold change ratio of ~0.9, fdr < 0.05). BLAST analysis did not reveal a conclusive functional prediction. However, closer inspection of the primary sequence showed amino acids in its C-terminal part that are conserved in the anti-sigma factor NepR (#1444)^17^. Some of these are key residues for protein-protein interaction with activated PhyR in *S. melonis* Fr1^17^. We additionally found that the predicted secondary structure of #1448 is similar to that of NepR (Fig. S5) according to YASPIN (http://www.ibi.vu.nl/programs/yaspinwww/)^76^.

**Figure 6:**
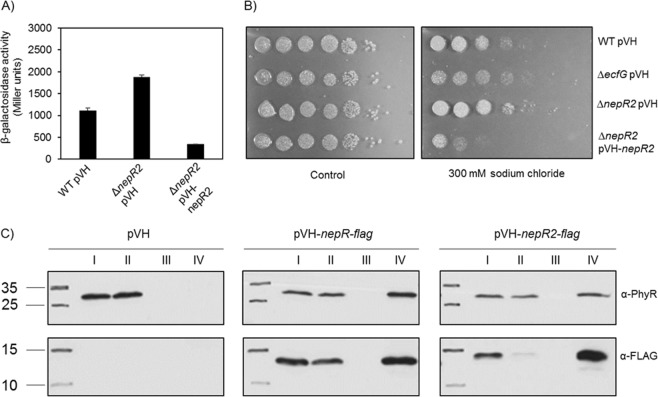
NepR2 is a negative feedback regulator of the GSR. (**A**) β-Galactosidase activity of the EcfG-dependent *nhaA2p-lacZ* reporter in *S. melonis* Fr1 wild type (WT) and a Δ*nepR2* mutant in low stress TYE medium after overnight overexpression of NepR2 from the vanillate-inducible pVH vector with 250 µM vanillate. pVH was used as empty vector control. Values are given as mean ± SD of three independent experiments. (**B**) Salt sensitivity assay with *S. melonis* Fr1 wild type (WT), a Δ*ecfG*, a Δ*nepR2* mutant, and the latter overexpressing NepR2 from the vanillate-inducible pVH vector with 250 µM vanillate. pVH was used as an empty vector control. The respective strains were grown in NB medium. OD_600_ was normalized to 1 prior to spotting 10-fold serial dilutions of each culture on NB agar plates with or without 300 mM sodium chloride. The pictures were taken 2-8 days after incubation at 28 °C and are representative of three independent biological replicates. (**C**) Co-immunoprecipitation using lysates originating from wild type either overexpressing NepR-Flag or NepR2-Flag from pVH or carrying the corresponding empty vector control. To allow binding of the Flag-tagged proteins, the lysates were incubated with the ANTI-FLAG M2 affinity gel. Samples were taken prior to incubation with the resin (sample I), from the supernatant after incubation (sample II), from the last wash of the resin (sample III), and from the ANTI-FLAG M2 eluate (sample IV) prior to analysis by non-reducing SDS-PAGE and Western blot analysis with a PhyR-antiserum (exposure time: 10 s) as well as an anti-Flag antibody (exposure time: 7 s). Results are representative of three independent biological replicates. Full-length blots are shown in Fig. S6.

Based on these findings, we further analyzed the role of this putative NepR-like regulator in the GSR. First, we performed β-galactosidase assays comparing GSR activity in the corresponding knockout mutant to the wild type (Fig. 6A). In addition, we tested the effect of overexpressing the putative regulator. The knockout of the NepR-like regulator increased GSR activity, while its overexpression in the knockout background reduced it even below wild-type level. These findings indicate that the protein indeed acts as a negative NepR-like GSR feedback regulator. To phenotypically validate our findings, we performed salt sensitivity assays (Fig. 6B). These indicated that overexpression of the putative regulator reduces resistance towards sodium chloride stress. Therefore, and due to the similarities with the anti-sigma factor NepR, we named the novel regulator NepR2. Binding of NepR2 to PhyR was confirmed by co-immunoprecipitation (Figs 6C, S6). We also tested an interaction of NepR2 and EcfG with BACTH assays (data not shown); however, both proteins were unstable when co-expressed. This is in contrast to what we observed for co-expression of complex-forming NepR and EcfG, which was tested as a control. This observation might suggest that NepR2 and EcfG do not form a complex. Such an interaction might have prevented protein degradation.

In additional β-galactosidase experiments, we tested the importance of individual selected amino acids (L32, R33, Y36 and M48) known to play a role in PhyR-NepR binding (Fig. S7)^17^ for the activity of NepR2. β-Galactosidase activity was measured in the Δ*nepR* and the Δ*nepR2* mutants overexpressing either NepR or NepR2 wild-type proteins or derivatives, in which one of the indicated amino acids was replaced by alanine. GSR induction was measured after exposure to the stress mix as described above. The negative regulatory function of NepR and NepR2 was decreased when L31 and F35 in NepR2 and L32 and Y36 in NepR were replaced by alanine, indicating that the functional importance of these amino acids is conserved. In contrast, M46 of NepR2, corresponding to M48 of NepR, did not change the activity of NepR2. The mutation of R32 in NepR2 and of R33 in NepR to alanine did not reduce activity of the respective regulators under the tested *in vivo* conditions.

Overall, our results suggest that NepR2 acts as a PhyR antagonist, as it has been suggested for MexAM1_meta2p0735 in *M. extorquens*^19^. The discovery of NepR2 as a negative feedback regulator illustrates another example of the ability of the GSR in *S. melonis* Fr1 to prevent an overactivation. In addition, it provides a means by which the GSR might be turned off after ceasing of stressful conditions.

## Conclusion

In this study, we identified the genes controlled by EcfG under low stress conditions in *S. melonis* Fr1. These include genes encoding proteins of various cellular processes such as metabolism, transport, envelope modulation, signal transduction and stress protection. However, a considerable proportion of the EcfG-regulated genes are of unknown function, representing opportunities to identify novel characteristics of the GSR. Our results indicate that the GSR adjusts to more severe stress conditions by increasing the promoter activity of EcfG-dependent genes and by enlarging the total number of genes controlled by the alternative sigma factor. In addition, we found that a stress mix, but not the individually tested stress stimuli, induces a transcriptional downregulation of genes encoding motility- and biofilm formation-associated proteins in *S. melonis* Fr1, and that this regulation depends on the single domain response regulator SdrG and the signal-integrating Paks. In the course of this study, we additionally identified and characterized the novel negative GSR feedback regulator NepR2. Altogether, our work emphasizes the complexity of the GSR regulatory network. It likely serves to optimize protein allocation and thus cellular resources, while ensuring survival and growth under fluctuating, stressful conditions.

## Experimental Procedures

### Mutant strain construction

The plasmid pAK405 was used for in-frame deletions in *S. melonis* Fr1 via double homologous recombination^77^. All gene numbers refer to Sphme2DRAFT available through https://img.jgi.doe.gov/m/.

### Plasmid construction

Table S9 lists all plasmids, which were constructed in the course of this study. The corresponding primers are deposited in Table S10. For construction of the vanillate-inducible expression plasmids pVH-*nepR2*, pVH-*nepR2*-*flag*, and pVH-*nepR*-*flag*, *nepR2* (#1448) for overexpression without a Flag-tag was amplified from *S. melonis* Fr1 genomic DNA with the primers “NepR2 XbaI fwd” and “NepR2 KpnI rev”, while *nepR2* with a Flag-tag was amplified with the primers “NepR2 XbaI fwd” and “NepR2 KpnI rev_2”. The same template was used for amplification of *nepR* (#1444) with a Flag-tag with the primers “NepR_Flag fwd XbaI” and “NepR_Flag rev KpnI”. The restriction enzymes KpnI and XbaI were used to digest the PCR products and the pVH backbone^78^ prior to ligation. For the construction of knockout plasmids, the primers “ EcfG HR1 fwd (BamHI)”, “EcfG HR1 rev”, “EcfG HR2 fwd”, and “EcfG HR2 rev (HindIII)” were used to amplify the up- and downstream regions (~750 bp) of *ecfG* (#1447) from *S. melonis* Fr1 genomic DNA. *NepR2* up- and downstream regions (#1448) were amplified from the same template with the primers “NepR2 HR1 fwd KpnI”, “NepR2 HR1 rev”, “NepR2 HR2 fwd” and “NepR2 HR2 rev HindIII”. An overlap PCR was used to join the up- and downstream regions prior to digestion with BamHI and HindIII for the *ecfG* knockout plasmid and with KpnI and HindIII for the *nepR2* knockout plasmid. The resulting inserts were ligated in the pAK405 backbone^77^. For construction of β-galactosidase reporter plasmids, the promoter regions of *#1102*, *#1276*, *#2499*, and *#3505* were amplified from *S. melonis* Fr1 genomic DNA with the primers “P1102_fwd “ and “P1102_rev”, “P1276_fwd “ and “P1276_rev”, “P2499_fwd” and “P2499_rev”, “P3505_fwd” and “P3505_rev”. After digestion with BamHI and HindIII, the inserts were ligated with the pAK501 backbone^16^.

### Site-directed mutagenesis of *nepR2* and *nepR*

L31, R32, F35, and M46 in *nepR2*-*flag* and L32, R33, Y36 and M48 in *nepR*-*flag* encoded on pVH^78^ were changed to alanine with site-directed mutagenesis using the QuikChange method (modified from Agilent Technologies). Briefly, the plasmids were amplified by Phusion High-Fidelity DNA Polymerase (Life Technologies) and digested with DpnI (New England Biolabs) prior to transformation in competent *E. coli* (DH5α). For *nepR2*-*flag* mutagenesis, we used the primers “NepR2 L31A_fwd” and “NepR2 L31A_rev”, “NepR2 R32A_fwd” and “NepR2 R32A_rev”, “NepR2 F35A_fwd” and “NepR2 F35A_rev”, and “NepR2 M46A_fwd” and “NepR2 M46A_rev”. For mutagenesis in *nepR*-*flag*, we used the primers “NepR L32A_fwd” and “NepR L32A_rev”, “NepR R33A_fwd” and “NepR R33A_rev”, “NepR Y36A_fwd” and “NepR Y36A_rev”, and “NepR M48A_fwd” and “NepR M48A_rev”.

### RNA sample preparation and sequencing

The *S. melonis* Fr1 wild-type strain and the Δ*ecfG* (*ecfG* #1447), Δ*phyR* (*phyR* #1445), Δ*sdrG* (*sdrG* #3354) mutants, as well as a complete Δ*pak* (*pakA* #2181, *pakB* #0513, *pakC* #2824, *pakD* #3653, *pakE* #2488, *pakF* #1661, *pakG* #3817) mutant were streaked out from cryo-stocks on TYE agar plates (1% tryptone, 0.5% yeast extract, 2.4 mM Na_2_HPO_4_, 37.6 mM KH_2_PO_4_, 1.5% agar agar) and were incubated overnight at 28 °C. All strains used in the RNA sequencing approach lack two of the four extrachromosomal plasmids and therefore the plasmid-encoded GSR-activating histidine kinase PakD and the single domain response regulator PkrD (#3654)^16^. These two extrachromosomal plasmids are frequently lost during the gene knockout procedure. The pre-cultures were inoculated in 20 mL TYE medium in 100 mL baffled flasks and were incubated during the day at 28 °C. The 225 mL overnight cultures were prepared in 1 L baffled flasks by diluting the pre-cultures to an OD_600_ so as to obtain exponentially growing cells at the time of the assay on the next day and were then incubated at 28 °C. For the wild-type strain and the Δ*ecfG* mutant, the bacterial cultures were divided into 5 × 40 mL cultures in 250 mL baffled flasks on the next day, after reaching exponential growth phase. Sodium chloride (80 mM) was added to the first flask, ethanol (1%) to the second, tert-butyl hydroperoxide (50 µM) to the third, a mix of the three stresses (stress mix) to the fourth, and the fifth was left untreated. 40 mL of the 225 mL overnight cultures of the other mutant strains were also transferred to 250 mL baffled flasks after reaching exponential phase on the next day. These cultures were treated with the stress mix. After further incubation of the cultures at 28 °C for 1 h, they were transferred to 50 mL Falcon tubes containing cold stop solution (5% phenol in ethanol, stored at −20 °C). After centrifugation (3220 g, 10 min, 4 °C), the supernatant was discarded and the pellets were frozen in liquid nitrogen and stored at −80 °C. Three independent biological replicates were prepared for RNA sequencing. The RNA was extracted with the RNeasy kit (Qiagen), after cell lysis with the Tissue Lyser (Qiagen/Retsch) using heat sterilized 0.1 mm zirconia beads (BioSpec). In addition to DNAse treatment on column (Qiagen), a second DNase treatment was performed after RNA preparation, using the Rapid out DNA removal kit (Thermo Scientific). RNasin® Plus RNase Inhibitor (Promega AG) was added to prevent RNA degradation. RNA quality was analyzed with the Agilent Tape Station/Bioanalyzer and concentration was determined with the Qubit fluorometer (Thermo Scientific) at the Functional Genomics Center Zurich (FGCZ). rRNA was depleted using the Ribo-Zero® rRNA Removal Kit (Bacteria) (Illumina, Inc.). Library preparation and mRNA sequencing (HiSeq 4000) were performed by the FGCZ.

### EcfG-binding motif identification

A gapped motif was constructed from the 72 sequences highlighted in reference^24^ as targets of EcfG in *B. diazoefficiens* using GLAM2^63^, part of the MEME Suite^64^, with default parameters other than increasing the number of alignment runs to 100. Regions 500 bp upstream of annotated coding sequences in the *S. melonis* Fr1 genome, except for the two extrachromosomal plasmids lacking in all strains, were extracted using a Python script (v2.7.12 with Biopython). The regions were truncated in case of overlap, treating each strand separately. This avoided cases in which the upstream region of a gene occurring downstream of a short gene might contain the same potential binding site as that of the short gene, so that sites would not be counted twice. The first gene encountered in the 5′-to 3′ direction has priority in such a case.

The extracted upstream regions were scanned for the gapped motif^24^ using GLAM2Scan^63^, returning the best scoring 10,000 hits. The results were then filtered using an R script (v3.3.1) to find the best scoring hit for each gene, recording the sequence, distance to the start of the coding sequence and score for each hit.

The top 50 highest scoring sequences for the genes differentially regulated by EcfG were then fed back into GLAM2 to construct a new gapped motif tailored to EcfG in *S. melonis* Fr1. This improved gapped motif was again scanned for in the same 500 bp upstream regions of *S. melonis* Fr1 as previously described using GLAM2Scan, returning the best scoring 10,000 hits. Once again, R was used to find the best scoring hit for each gene. The motif scoring provided in Table 1 and Table S3 indicates a log-likelihood ratio between the motif and a background model, i.e. a difference of 1 between the two scores means that the higher scoring sequence is twice as likely as the lower scoring.

### β-Galactosidase assays

The *S. melonis* Fr1 strains carrying the indicated reporter plasmids were streaked out from cryo-stocks on TYE agar plates containing chloramphenicol (34 µg mL^−1^) and were incubated overnight at 28 °C. TYE pre-cultures (20 mL) supplemented with chloramphenicol (34 µg mL^−1^) were inoculated in 100 mL baffled flasks and were further incubated at 28 °C during the day. TYE overnight cultures (240 mL) grown in 1 L baffled flasks and 20 mL TYE overnight cultures grown in 100 mL baffled flasks were supplemented with chloramphenicol (34 µg mL^−1^) and were inoculated in the evening at an OD_600_ so as to obtain exponentially growing cells at the time of the assay on the next day. For the GSR activation with different stress stimuli, the 240 mL overnight cultures were split into 20 mL cultures as soon as the cells reached exponential growth phase. The bacteria were immediately exposed to the respective stress stimuli, while one flask was left untreated. The β-galactosidase assays (Miller 1972) were performed 1 h after exposure to the stress stimuli. All assays were carried out in three independent biological replicates. For β-galactosidase assays with overexpression of NepR2 or NepR, TYE agar plates and culture medium used for the strains carrying pVH expression plasmids were supplemented with tetracycline (10 µg mL^−1^) in addition to chloramphenicol (34 µg mL^−1^). The β-galactosidase assays were performed with the *nhaA2p-lacZ* fusion^16^. The bacteria were streaked out from cryo-stocks on TYE agar plates and 20 mL TYE pre-cultures were grown in 100 mL baffled flasks at 28 °C. For overexpression, the 20 mL overnight cultures of *S. melonis* Fr1 grown in 100 mL baffled flasks, were diluted from pre-cultures at an OD_600_ so as to obtain exponentially growing cells at the time of the assay on the next day. The overnight cultures were supplemented with vanillate (250 µM final concentration, 250 mM stock in 100% ethanol) in addition to the appropriate antibiotics indicated above. pVH was used as an empty vector control. The measurements (Miller 1972) depicted in Fig. 6A were carried out when bacteria reached exponential growth phase without exposure to a stress stimulus. The β-galactosidase assays (Miller 1972) shown in Fig. S7 were performed 1 h after exposure to the stress mix. Three independent biological replicates were performed for all experiments.

### Salt sensitivity assays

The protocol for the salt sensitivity assays was adopted from^43^ with minor changes. The respective strains, which carried the empty pVH vector as a control or pVH-*nepR2* together with the *nhaA2p-lacZ* fusion, were streaked out from cryo-stocks on NB (Sigma-Aldrich) agar plates, which were supplemented with tetracycline (10 µg mL^−1^) and incubated at 28 °C overnight. NB pre-cultures (20 mL) supplemented with tetracycline (10 µg mL^−1^) were inoculated in 100 mL baffled flasks and incubated during the day at 28 °C. Overnight cultures (20 mL) supplemented with tetracycline (10 µg mL^−1^) and vanillate (250 µM) were inoculated in 100 mL baffled flasks at an OD_600_ so as to obtain exponentially growing cells at the time of the assay on the next day and were incubated at 28 °C. After reaching exponential growth phase, 1 mL of each culture was spun down (3000 g, 5 min) and re-suspended in NB medium to an OD_600_ of 1. After preparing 10-fold dilution series in NB medium, 4 µL of each dilution were spotted on NB agar plates supplemented with tetracycline (10 µg mL^−1^) and vanillate (250 µM). In addition, 300 mM sodium chloride were added to one plate, but not to the control plate. The plates were sealed with parafilm. Pictures were taken after incubation at 28 °C for 2–8 days. The assay was repeated in three independent biological replicates.

### Co-immunoprecipitation

The *S. melonis* Fr1 wild-type strain carrying pVH-*nepR2*-*flag* (*nepR2* #1448), pVH-*nepR*-*flag* (*nepR* #1444), or pVH as the empty vector control, were streaked out from cryo-stocks on TYE agar plates supplemented with tetracycline (10 µg mL^−1^) and incubated overnight at 28 °C. The pre-cultures were inoculated in 20 mL TYE medium containing tetracycline (10 µg mL^−1^) in 100 mL baffled flasks. The overnight cultures were inoculated from pre-cultures in 100 mL TYE medium supplemented with tetracycline (10 µg mL^−1^) and vanillate (250 µM) in 500 mL baffled flasks at an OD_600_ so as to reach exponential growth phase at the time point of the assay on the next day. The co-immunoprecipitation was carried out as previously described with minor modifications^19^. The bacteria were harvested at an OD_600_ of 1 via centrifugation (4000 g, 4 °C, 15 min) and washed once with 10 mL TBS buffer (50 mM Tris pH 7.4, 150 mM NaCl). Afterwards, the bacteria were re-suspended in 0.5 mL TBS buffer supplemented with EDTA-free Complete Protease Inhibitor (Roche Diagnostics), DNaseI (Roche Diagnostics), and lysozyme (1 mg mL^−1^, Sigma Aldrich). Subsequently, the cells were lysed using the CapMix^TM^ (3 M ESPE AG) (3 × 30 sec) with 0.1 mm zirconia beads (BioSpec). After centrifugation (12.000 g, 4 °C, 10 min), the supernatant was transferred to a new tube. This centrifugation step was repeated once. Per sample, 40 µL of ANTI-FLAG M2 affinity gel (Sigma-Aldrich) were washed four times with 1 mL TBS buffer before being re-suspended in 20 µL TBS buffer supplemented with Complete Protease Inhibitor EDTA-free (Roche Diagnostics). Before adding the bacterial supernatant to 20 µL ANTI-FLAG M2 affinity gel, 50 µL were stored separately for Western blot analysis (sample I). Next, the mixture was incubated for 1 h at room temperature under gentle agitation. After centrifugation (6000 g, 30 sec), 50 µL of the supernatant were taken for Western blot analysis (sample II). The resin was washed four times with 0.5 mL TBS buffer supplemented with protease inhibitor. 50 µL of the last wash were taken for Western blot analysis (sample III). Afterwards, the resin was re-suspended in 50 µL 2.5x non-reducing SDS-PAGE loading buffer (2 ml 1.5 M Tris-HCl pH 6.8, 5 ml glycerol, 1 g SDS, 0.5 ml 1% bromphenol blue, 10 ml ddH_2_O) and boiled for 5 min. Following centrifugation (6000 g, 30 s), the supernatant was taken as sample IV. All samples were subjected to SDS-PAGE for Western blot analysis using a rabbit PhyR-antiserum (1:10.000) (BioGenes)^15^, as well as a mouse anti-Flag antibody (1:4000) (Sigma-Aldrich). In addition, HRP-conjugated goat anti-rabbit (1:5000) and goat anti-mouse (1:3000) (Biorad) secondary antibodies were used.

## Supplementary information

Supplementary Information

Table S1

Table S2

Table S3

Table S4

Table S5

Table S6

Table S7

Table S8

## Data Availability

The RNA sequencing data will be available at the European Nucleotide Archive under the accession number PRJEB30300.
