# Supplementary material for: Complex general stress response regulation in Sphingomonas melonis Fr1 revealed by transcriptional analyses
Source: Sci Rep. 2019 Jun 28;9:9404. doi: 10.1038/s41598-019-45788-7 (PMC6599016; doi:10.1038/s41598-019-45788-7)
Supplement: Supplementary file 1 — Supplementary Information [file 41598_2019_45788_MOESM1_ESM.pdf]

**Supplementary Information**

# Complex general stress response regulation in *Sphingomonas melonis* Fr1 revealed by transcriptional analyses

Lisa Gottschlich[1], Petra Geiser[1], Miriam Bortfeld-Miller[1], Christopher M. Field[1], Julia A. Vorholt[1*]

[1]Institute of Microbiology, Department of Biology, ETH Zurich, Vladimir-Prelog-Weg 1-5/10, 8 8093 Zurich, Switzerland

*Correspondence: jvorholt@ethz.ch

**Figures S1-7**

**Table legends S1-8**

**Tables S9-10**

**References**

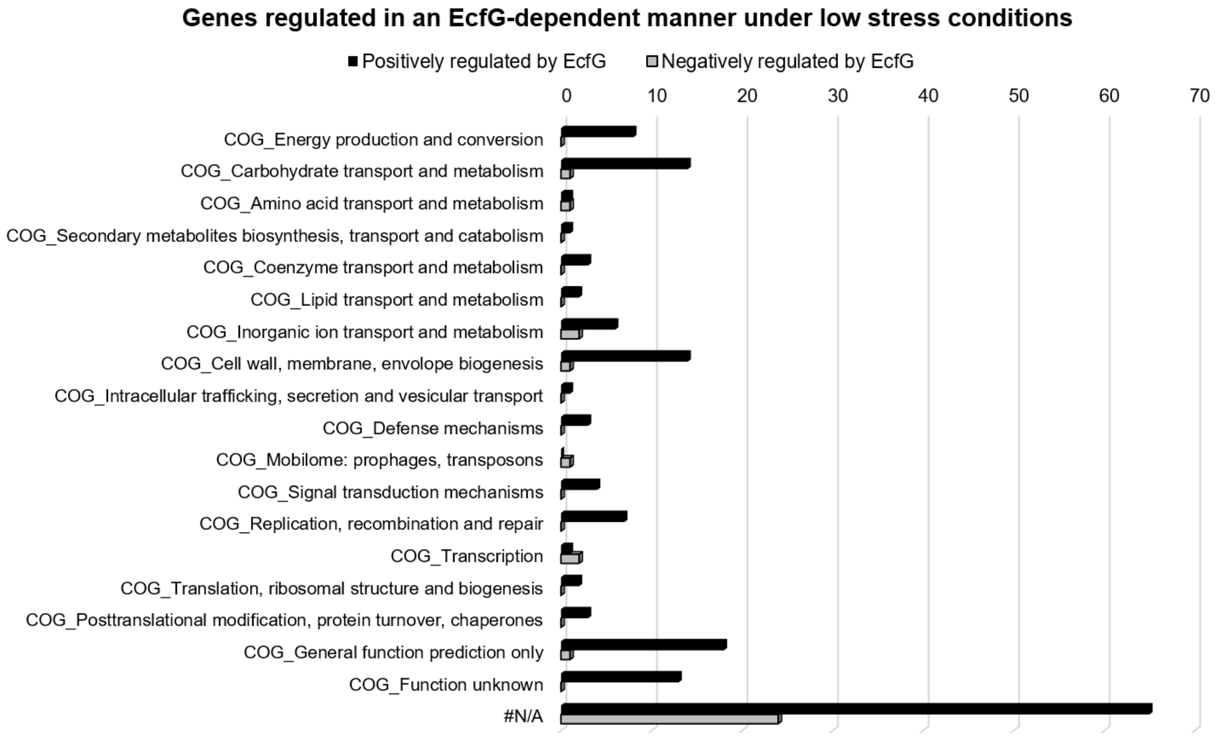


**Figure S1. Genes regulated by EcfG under low stress conditions.** Clusters of Orthologous Groups (COG) categories[1] were retrieved from the Integrated Microbial Genomes with Microbiome Samples system (IMG/M: https://img.jgi.doe.gov/m/)[2]. Genes transcriptionally up- (grey) or downregulated (black) (cutoff: $\log_2$ fold change ratio < [-1] and >1, fdr <0.05) in the *S. melonis* Fr1 Δ*ecfG* mutant compared to the wild-type strain under low stress conditions (TYE medium without stress induction) are shown (Table S2). Transcriptome analysis was performed with three independent biological replicates.

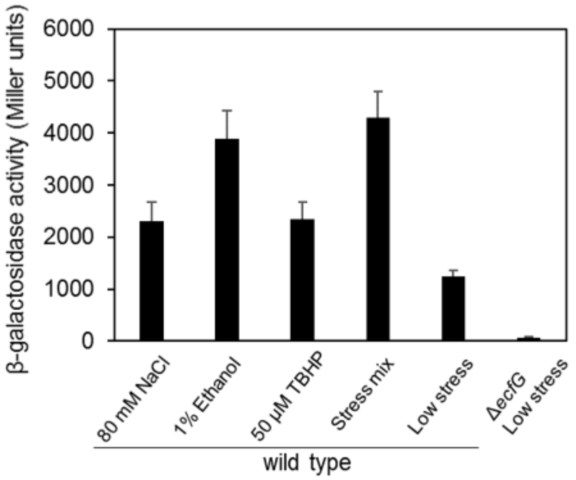


**Figure S2. GSR activation under different stress conditions.** β-Galactosidase activity of the *nhaA2p-lacZ* fusion in *S. melonis* Fr1 wild type and the Δ*ecfG* mutant 1 h after exposure to the indicated stress conditions. The stress mix consists of a combination of the indicated single stresses and low stress conditions are represented by growth in TYE medium. Values are given as mean ±SD of three independent experiments.

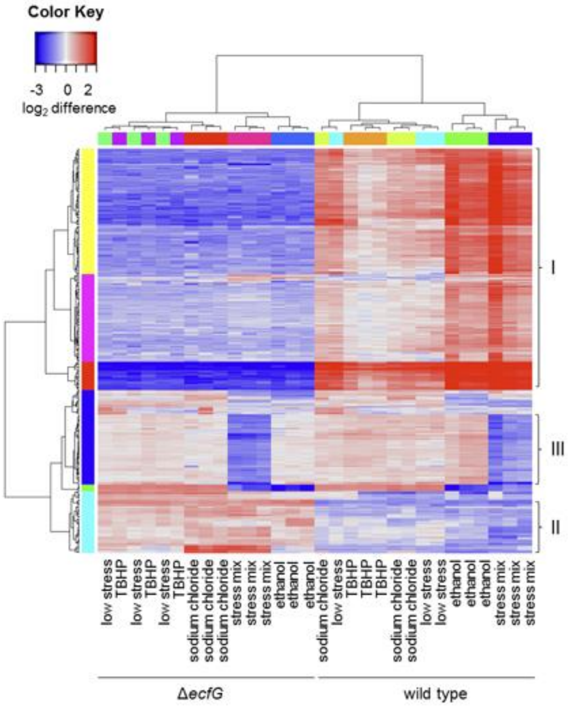


**Figure S3. The effects of different stresses on the transcriptome.** Heatmap comparing the 300 most variable genes of *S. melonis* Fr1 wild type and the Δ*ecfG* mutant under low stress conditions (TYE medium) and 1 h after exposure to 80 mM sodium chloride, 1% ethanol, 50 µM tert-butyl hydroperoxide (TBHP) or a combination of the three stresses (stress mix) (Table S5). Clusters I and II represent the genes which are up- or downregulated depending on the *ecfG* knockout. Cluster III contains mainly motility- and biofilm formation-associated genes. These are downregulated in an EcfG-independent manner after exposure to the stress mix. The heatmap was generated with the function heatmap.2 from the R-package "gplots" (version 3.0.1). The image represents three independent biological replicates.

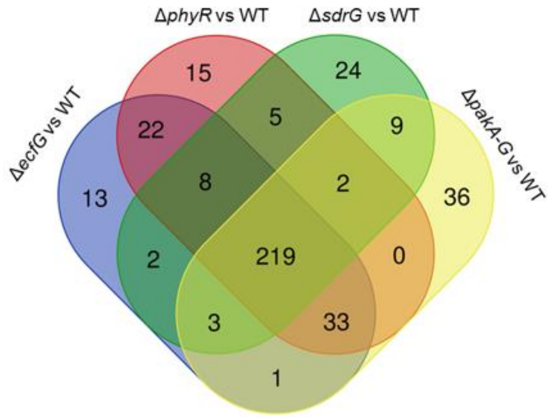


**Figure S4. The EcfG-controlled genes are congruent with those controlled by PhyR, SdrG, and the Paks.** Venn diagram modified from the UGent Venn diagram web tool (http://bioinformatics.psb.ugent.be/webtools/Venn/). It compares genes significantly regulated in the indicated *S. melonis* Fr1 key regulator knockout mutants (three biological replicates) compared to the wild type (cutoff: $\log_2$ fold change ratio < [-1] and > 1, fdr < 0.05) 1 h after exposure to the stress mix (80 mM sodium chloride, 1% ethanol, 50 µM tert-butyl hydroperoxide). EcfG controls 301 genes, PhyR controls 304 genes, SdrG controls 272 genes, and the Paks control 303 genes. The knocked-out genes also appear as regulated genes (Table S7).

```
NepR2  MNANAHPVEKSRQGDYVVRRPRSTDALGHSLRGAFGSSDMPDDFAMLLKRIDRATH
       ----------------EEEE----HHHHHHHHH--------HHHHHHHHHHHH---

NepR   MLDLPGNKDKKASSKKSPAKVQSKDRDMGAALRSAYQKTIEEQVPDEMLDLLNKLA
       ------------------EEE-----HHHHHHHHHHHHHHHHH--HHHHHHHHHH-
```

**Figure S5. Similarities between NepR and NepR2.** Comparison of the amino acid sequences and secondary structures of NepR (#1444) and NepR2 (#1448) reveals similarities between both proteins. The amino acids highlighted in red have been tested for functionality in β-galactosidase assays (Fig. S7). They belong to the amino acids which are important for the NepR-PhyR interaction [3]. The secondary structures of NepR and NepR2, predicted with the prediction tool YASPIN (http://www.ibi.vu.nl/ programs/yaspinwww/), are depicted below the corresponding amino acid sequence. 'H' stands for 'helix', 'E' means 'strand' and '-' represents 'other' [4].

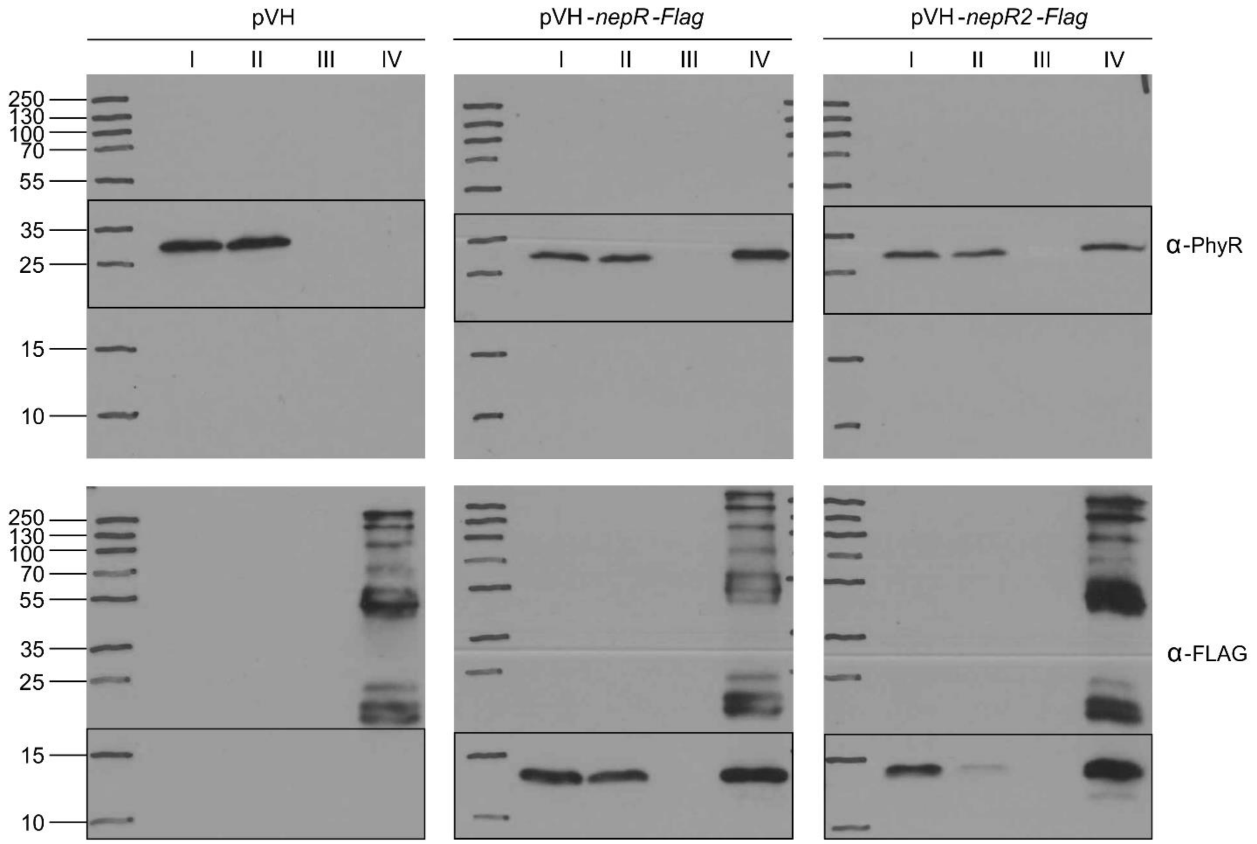


**Figure S6. Co-immunoprecipitation of NepR and NepR2.** A PhyR-antiserum (exposure time: 10 s) and an anti-Flag antibody (exposure time: 7 s) were used for Western blotting, following non-reducing SDS-PAGE. Sample I: prior to incubation with the resin. Sample II: Supernatant after incubation. Sample III: Last wash of the resin. Sample IV: ANTI-FLAG M2 eluate. Results are representative of three independent biological replicates, for further details see Fig. 6. The black rectangles indicate the cropped areas shown in Fig. 6.

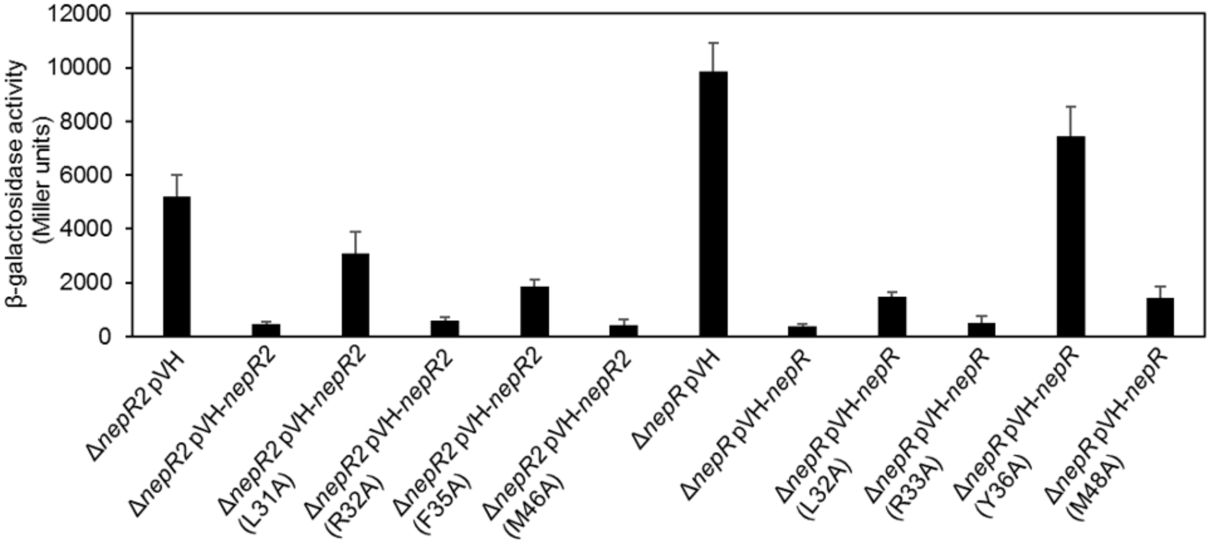


**Figure S7. Identification of amino acids important for the negative regulatory function of NepR2.** β-Galactosidase activity of the EcfG-dependent *nhaA2p-lacZ* fusion in the *S. melonis* Fr1 Δ*nepR* and Δ*nepR2* mutants 1 h after exposure to the stress mix (80 mM sodium chloride, 1% ethanol, 50 µM tert-butyl hydroperoxide) after overnight overexpression of NepR or NepR2 from the vanillate-inducible pVH vector with 250 µM vanillate. pVH was used as empty vector control. Values are given as mean ±SD of three independent experiments.

**Supplementary Information Table Legends S1-8**

**Table S1.** Genes regulated in an EcfG-dependent manner under low stress conditions. Transcript IDs, predicted annotation, $\log_2$ fold change ratios resulting from the Δ*ecfG* mutant vs wild type (WT) (low stress) comparison and the corresponding fdr values are indicated.

**Table S2.** Data corresponding to Fig. S1, representing the classification of the proteins encoded by the genes regulated by EcfG under low stress conditions with COG categories. $\text{Log}_2$ fold change ratios and the fdr values for the Δ*ecfG* mutant vs wild type (WT) comparison under low stress conditions are also indicated.

**Table S3.** Results of the genome-wide screen for the EcfG-binding motif specific for *S. melonis* Fr1. Columns B and C indicate the presence (TRUE) or absence (FALSE) of the respective genes regulated by EcfG under low stress conditions or 1 h after exposure to the stress mix (cutoff: $\log_2$ fold change ratio < [-1] and > 1, fdr < 0.05). The sequence containing the putative EcfG-binding motif is given, as well as its distance to the start of the predicted coding sequence and its score. Significantly positively regulated genes are also indicated as putative direct EcfG targets with an "X", if they are located within an optimum distance of about 100 bp upstream of the start of the predicted CDS and if their motif score is ≥ 10. The associate COG category is indicated as well as the $\log_2$ fold change ratios with fdr values for the following comparisons: Δ*ecfG* mutant vs wild type (WT) (low stress), Δ*ecfG* mutant vs WT (stress mix), WT (stress mix vs low stress), Δ*ecfG* mutant (stress mix vs low stress). For further details see Experimental Procedures section.

**Table S4.** Data corresponding to Fig. 4, representing the comparison of the genes controlled by EcfG under low stress conditions and 1 h after exposure to the stress mix with a Venn diagram

(http://bioinformatics.psb.ugent.be/webtools/Venn/) and a scatter plot. The transcript IDs of the genes regulated by the stress mix in the Δ*ecfG* mutant are highlighted in yellow. $Log_2$ fold change ratios and the fdr values for the following comparisons are indicated: Δ*ecfG* mutant vs wild type (WT) (low stress), Δ*ecfG* mutant vs WT (stress mix), WT (stress mix vs low stress), Δ*ecfG* mutant (stress mix vs low stress). The input data for the Venn diagram is given.

**Table S5.** Data corresponding to the heatmap represented in Fig. S3, including $\log_2$ fold change ratios and the fdr values for the wild type (WT) and the Δ*ecfG* mutant 1 h after exposure to different stress stimuli (80 mM sodium chloride (NaCl), 1% ethanol (EtOH), 50 µM tert-butyl hydroperoxide (TBHP) and a mix of the three stress stimuli (stress mix)) compared to the low stress samples, which were left untreated.

**Table S6.** Data corresponding to the heatmap represented in Fig. 5, including $\log_2$ fold change ratios and the fdr values for the comparisons of the GSR key regulator mutants (Δ*ecfG,* Δ*phyR,* Δ*sdrG,* complete Δ*pak* mutant) with the wild type (WT) 1 h after exposure to the stress mix (80 mM sodium chloride, 1% ethanol, 50 µM tert-butyl hydroperoxide).

**Table S7.** Data corresponding to Fig. S4, representing a Venn diagram comparison of the genes regulated by the GSR key regulators EcfG, PhyR, SdrG, and the Paks 1 h after exposure to the stress mix (80 mM sodium chloride, 1% ethanol, 50 µM tert-butyl hydroperoxide). The $\log_2$ fold change ratios and the fdr values for all comparisons of the respective mutant strains vs wild type (WT), as well as the input data for the Venn diagram are given.

**Table S8.** Data corresponding to the comparison of the genes present in cluster III in Fig. 5 and in cluster III in Fig. S3, which was made with the help of the UGent Venn diagram web tool (http://bioinformatics.psb.ugent.be/webtools/Venn/). The $\log_2$ fold change ratios and the fdr values

for the Δ*sdrG* mutant vs wild type (WT) comparison 1 h after exposure to the stress mix (80 mM sodium chloride, 1% ethanol, 50 µM tert-butyl hydroperoxide) and the comparison of the wild type (WT) 1 h after exposure to the stress mix vs low stress conditions are given.

**Table S9.** List of all plasmids and strains used in this study.

| Plasmids | Genotype and/or relevant features | Reference or source |
|---|---|---|
| pAK405 | Plasmid for markerless gene deletion; kan$^r$ | [5] |
| pAK405-*nepR2* | Plasmid for markerless deletion of *nepR2*; kan$^r$ | This study |
| pAK405-*ecfG* | Plasmid for markerless deletion of *ecfG;* kan$^r$ | This study |
| pVH; tet$^r$ | Plasmid for vanillate-inducible protein expression; tet$^r$ | [6] |
| pVH-*nepR-flag* | Plasmid for vanillate-inducible production of HA-NepR-Flag; tet$^r$ | This study |
| pVH-*nepR-flag* (L32A) | Plasmid for vanillate-inducible production of HA-NepR-Flag (L32A); tet$^r$ | This study |
| pVH-*nepR-flag* (R33A) | Plasmid for vanillate-inducible production of HA-NepR-Flag (R33A); tet$^r$ | This study |
| pVH-*nepR-flag* (Y36A) | Plasmid for vanillate-inducible production of HA-NepR-Flag (Y36A); tet$^r$ | This study |
| pVH-*nepR-flag* (M48A) | Plasmid for vanillate-inducible production of HA-NepR-Flag (M48A); tet$^r$ | This study |
| pVH-*nepR2* | Plasmid for vanillate-inducible production of HA-NepR2; tet$^r$ | This study |
| pVH-*nepR2-flag* | Plasmid for vanillate-inducible production of HA-NepR2-Flag; tet$^r$ | This study |
| pVH-*nepR2-flag* (L31A) | Plasmid for vanillate-inducible production of HA-NepR2-Flag (L31A); tet$^r$ | This study |
| pVH-*nepR2-flag* (R32A) | Plasmid for vanillate-inducible production of HA-NepR2-Flag (R32A); tet$^r$ | This study |
| pVH-*nepR2-flag* (F35A) | Plasmid for vanillate-inducible production of HA-NepR2-Flag (F35A); tet$^r$ | This study |
| pVH-*nepR2-flag* (M46A) | Plasmid for vanillate-inducible production of HA-NepR2-Flag (M46A); tet$^r$ | This study |

| | | |
|---|---|---|
| pAK501-*pnhaA2-lacZ* | Reporter plasmid harboring a *nhaA2* promoter-*lacZ* transcriptional fusion; $cm^r$ | [7] |
| pAK501-*p1102-lacZ* | Reporter plasmid harboring a *sphme2DRART_1102* promoter-*lacZ* transcriptional fusion; $cm^r$ | This study |
| pAK501-*p1276-lacZ* | Reporter plasmid harboring a *sphme2DRAFT_1276* promoter-*lacZ* transcriptional fusion; $cm^r$ | This study |
| pAK501-*p2499-lacZ* | Reporter plasmid harboring a *sphme2DRAFT_2499* promoter-*lacZ* transcriptional fusion; $cm^r$ | This study |
| pAK501-*p3505-lacZ* | Reporter plasmid harboring a *sphme2DRAFT_3505* promoter-*lacZ* transcriptional fusion; $cm^r$ | This study |
| $kan^r$, kanamycin resistance; $amp^r$, ampicillin resistance $carb^r$, carbenicillin resistance; $tet^r$ tetracycline resistance.; $cm^r$ chloramphenicol resistance | | |
| | | |
| ***Escherichia coli* strains** | | |
| DH5α | *fhuA2 lac* (Δ)*U169 phoA glnV44 ϕ80' lacZ* (Δ)*M15 gyrA96 recA1 relA1 endA1 thi-1 hsdR17* | Invitrogen |
| ***Sphingomonas melonis* Fr1 strains** | | |
| JVZ857 | *Sphingomonas melonis* Fr1 WT strain | [8] |
| JVZ 4557 | JVZ857 lacking 2 extrachromosomal plasmids, therefore missing *pakD* and *pkrD* | [9] |
| JVZ4581 | JVZ4557 Δ*phyR* | [9] |
| JVZ4584 | JVZ4557 Δ*sdrG* | [9] |
| JVZ4556 | JVZ857 Δ*pakC*Δ*pakB*Δ*pakE*Δ*pakF*Δ*pakG* Δ*pakA* lacking 2 extrachromosomal plasmids, therefore missing *pakD* and *pkrD* | [9] |
| JVZ4558 | JVZ857 Δ*ecfG* lacking 2 extrachromosomal plasmids, therefore missing *pakD* and *pkrD* | This study |
| JVZ4559 | JVZ857 Δ*nepR2* | This study |
| JVZ2896 | Δ*nepR* | Francez-Charlot (unpublished) |

**Table S10.** List of all primers used in this study.

| Primer name | Sequence (5' -3') | Description |
|---|---|---|
| **Oligonucleotides for plasmid construction** | | |
| NepR_Flag fwd XbaI | AATTTCTAGAGATGTTGGATTTGCCC GGCAACAAG | *nepR-flag* in pVH |
| NepR_Flag rev KpnI | AATTGGTACCGCGGCGAGCTTGTTCA GCAGGTC | *nepR-flag* in pVH |
| NepR2 XbaI fwd | AATTTCTAGAGATGAACGCCAATGCG CATCCC | *nepR2* and *nepR2-flag* in pVH |
| NepR2 KpnI rev | AATTGGTACCTCAATGTGTCGCGCGG TCAA | *nepR2* in pVH |
| NepR2 KpnI rev_2 | AATTGGTACCGCATGTGTCGCGCGGT CAA | *nepR2-flag* in pVH |
| EcfG HR1 fwd (BamHI) | AAATTGGATCCTATCTGGGCGTCGGC CAATG | Knockout of *ecfG* |
| EcfG HR1 rev | CGCGGGTCGCTTCAGCGCATGATCAT CAAACCCCCGAAAC | Knockout of *ecfG* |
| EcfG HR2 fwd | CGCTGAAGCGACCCGCGGCTAGTG | Knockout of *ecfG* |
| EcfG HR2 rev (HindIII) | AATTAAGCTTCGCCTCTTCCGGCTCC TCACG | Knockout of *ecfG* |
| NepR2 HR1 fwd KpnI | AAATTGGTACCTTATCACCAGCCGCT ACCC | Knockout of *nepR2* |
| NepR2 HR1 rev | GGCGTTCATAATCGTTCCAACGG | Knockout of *nepR2* |
| NepR2 HR2 fwd | TTGGAACGATTATGAACGCCCATTGA TTGCGGCACTAGC | Knockout of *nepR2* |
| NepR2 HR2 rev HindIII | AGCAAGCTTATTTGCCGGAAGAGACA CC | Knockout of *nepR2* |
| P1102_fwd | AAATTTGGATCCCGAGGGCTTTCCGT TGCGGA | *p1102* in pAK501 |
| P1102_rev | AATTAAGCTTCAGCGCGAAGGGATTG TCGG | *p1102* in pAK501 |
| P1276_fwd | AAATTTGGATCCATGTGTGCGTGCGA GAAC | *p1276* in pAK501 |
| P1276_rev | AATTAAGCTTGCCGGGCAAGTGATCC TCC | *p1276* in pAK501 |
| P2499_fwd | AATTAAGCTTGATCGATGCCTTGGTG ATGG | *p2499* in pAK501 |
| P2499_rev | AAATTTGGATCCAAGAAACGAATGGG GTTCGG | *p2499* in pAK501 |
| P3505_fwd | AATTAAGCTTAATGGATCAATAGTCGC ACG | *P3505* in pAK501 |
| P3505_rev | AAATTTGGATCCCTCAACGACAGTTT GGTCG | *P3505* in pAK501 |
| NepR L32A_fwd | GGCCGCCCGCTCGGCCTATCAAAAG ACG | Mutagenesis of L32 in *nepR* |

| | | |
|---|---|---|
| NepR L32A_rev | CCTCGATCGTCTTTTGATAGGCCGAG CGGGCG | Mutagenesis of L32 in *nepR* |
| NepR R33A_fwd | ATGGGCGCGGCCCTGGCCTCGGCCT ATCAAAAG | Mutagenesis of R33 in *nepR* |
| NepR R33A_rev | TTTGATAGGCCGAGGCCAGGGCCGC GCCCATG | Mutagenesis of R33 in *nepR* |
| NepR Y36A_fwd | GGCCCTGCGCTCGGCCGCCCAAAAG ACGATCGAGGAAC | Mutagenesis of Y36 in *nepR* |
| NepR Y36A_rev | GTTCCTCGATCGTCTTTTGGGCGGCC GAGCGCAGGG | Mutagenesis of Y36 in *nepR* |
| NepR M48A_fwd | GAACAGGTGCCGGACGAAGCCCTCG ACCTGCTGAACAAGCTC | Mutagenesis of M48 in *nepR* |
| NepR M48A_rev | GAGCTTGTTCAGCAGGTCGAGGGCTT CGTCCGGCACCTGTTC | Mutagenesis of M48 in *nepR* |
| NepR2 L31A_fwd | ACTCGGCCACAGCGCCCGCGGCGCG TTCGGCAG | Mutagenesis of L31 in *nepR2* |
| NepR2 L31A_rev | TGCCGAACGCGCCGCGGGCGCTGTG GCCGAGTG | Mutagenesis of L31 in *nepR2* |
| NepR2 R32A_fwd | GCCACAGCCTGGCCGGCGCGTTCGG CAGC | Mutagenesis of R32 in *nepR2* |
| NepR2 R32A_rev | GCCGAACGCGCCGGCCAGGCTGTGG CCGAG | Mutagenesis of R32 in *nepR2* |
| NepR2 F35A_fwd | AGCCTGCGCGGCGCGGCCGGCAGCT CCGAC | Mutagenesis of F35 in *nepR2* |
| NepR2 F35A_rev | CATGTCGGAGCTGCCGGCCGCGCCG CGCAGG | Mutagenesis of F35 in *nepR2* |
| NepR2 M46A_fwd | CATGCCCGATGACTTCGCGGCCCTG CTAAAGCGGATTGAC | Mutagenesis of M46 in *nepR2* |
| NepR2 M46A_rev | CGGTCAATCCGCTTTAGCAGGGCCG CGAAGTCATCGGGCATGTC | Mutagenesis of M46 in *nepR2* |

## References


1. Tatusov, R. L., Koonin, E. V. & Lipman, D. J. A genomic perspective on protein families. *Science* **278**, 631-637, (1997).
2. Chen, I. A. *et al.* IMG/M: integrated genome and metagenome comparative data analysis system. *Nucleic Acids Res.* **45**, D507-D516, (2017).
3. Campagne, S. *et al.* Structural basis for sigma factor mimicry in the general stress response of Alphaproteobacteria. *Proc. Natl. Acad. Sci. U S A* **109**, E1405-1414, (2012).
4. Lin, K., Simossis, V. A., Taylor, W. R. & Heringa, J. A simple and fast secondary structure prediction method using hidden neural networks. *Bioinformatics* **21**, 152-159, (2005).
5. Kaczmarczyk, A., Vorholt, J. A. & Francez-Charlot, A. Markerless gene deletion system for Sphingomonads. *Appl. Environ. Microbiol.* **78**, 3774-3777, (2012).
6. Kaczmarczyk, A., Vorholt, J. A. & Francez-Charlot, A. Synthetic vanillate-regulated promoter for graded gene expression in *Sphingomonas*. *Sci. Rep.* **4**, 6453, (2014).
7. Kaczmarczyk, A., Hochstrasser, R., Vorholt, J. A. & Francez-Charlot, A. Complex two-component signaling regulates the general stress response in Alphaproteobacteria. *Proc. Natl. Acad. Sci. U S A* **111**, E5196-5204, (2014).
8. Innerebner, G., Knief, C. & Vorholt, J. A. Protection of *Arabidopsis thaliana* against leaf-pathogenic *Pseudomonas syringae* by *Sphingomonas* strains in a controlled model system. *Appl. Environ. Microbiol.* **77**, 3202-3210, (2011).
9. Gottschlich, L., Bortfeld-Miller, M., Gäbelein, C., Dintner, S. & Vorholt, J. A. Phosphorelay through the bifunctional phosphotransferase PhyT controls the general stress response in an alphaproteobacterium. *PLoS Genet.* **14**, e1007294, (2018).
